# Animal and Human Brucellosis in Pakistan

**DOI:** 10.3389/fpubh.2021.660508

**Published:** 2021-07-30

**Authors:** Tariq Jamil, Aman Ullah Khan, Muhammad Saqib, Muhammad Hammad Hussain, Falk Melzer, Abdul Rehman, Muhammad Zubair Shabbir, Mumtaz Ali Khan, Shahzad Ali, Asim Shahzad, Iahtasham Khan, Mudassar Iqbal, Qudrat Ullah, Waqas Ahmad, Muhammad Khalid Mansoor, Heinrich Neubauer, Stefan Schwarz

**Affiliations:** ^1^Institute of Bacterial Infections and Zoonoses, Friedrich-Loeffler-Institut, Jena, Germany; ^2^Institute of Microbiology and Epizootics, Freie Universität Berlin, Berlin, Germany; ^3^Section of Microbiology, Department of Pathobiology, University of Veterinary and Animal Sciences, Lahore, Pakistan; ^4^Department of Clinical Medicine and Surgery, Faculty of Veterinary Science, University of Agriculture, Faisalabad, Pakistan; ^5^Independent Researcher, Bardia, NSW, Australia; ^6^Department of Epidemiology and Public Health, Faculty of Veterinary Science, University of Veterinary and Animal Sciences, Lahore, Pakistan; ^7^Institute of Microbiology, University of Veterinary & Animal Sciences, Lahore, Pakistan; ^8^Field Epidemiology and Disease Survillence Division, National Institute of Health (NIH), Islamabad, Pakistan; ^9^Wildlife Epidemiology and Molecular Microbiology Laboratory (One Health Research Group), Discipline of Zoology, Department of Wildlife & Ecology, University of Veterinary and Animal Sciences, Lahore, Pakistan; ^10^Faculty of Veterinary and Animal Sciences, The Islamia University of Bahawalpur, Bahawalpur, Pakistan; ^11^Section of Epidemiology and Public Health, Department of Clinical Sciences, University of Veterinary and Animal Sciences, Lahore, Pakistan; ^12^Faculty of Veterinary and Animal Sciences, Gomal University, Dera Ismail Khan, Pakistan; ^13^Department of Clinical Sciences, University College of Veterinary and Animal Sciences, Narowal, Pakistan

**Keywords:** brucellosis, epidemiology, zoonosis, One Health, diagnosis, Pakistan

## Abstract

Brucellosis is a bacterial zoonotic disease that affects many animal species and can be transmitted to humans *via* direct contact or *via* contaminated food. Although brucellosis is a serious health hazard, its public health concern has been neglected in many countries. In some developing countries, such as Pakistan, where brucellosis is endemic, this disease continues to be of importance. A literature search for the past 11 years (2011–2021) provided a comprehensive insight into brucellosis in Pakistan. In this review, particular emphasis was placed on occurrence, diagnostic tests used, and prevention, treatment, and control in the context of the “One Health” approach.

## Introduction

Brucellosis is an important zoonotic disease of domestic livestock and wildlife caused by bacteria of the genus *Brucella* (*B*.). These bacteria are intracellular, Gram-negative, non-motile, non-spore-forming coccobacilli. Brucellae demonstrate animal host preferences, e.g., *B. melitensis* primarily infects small ruminants, *B. abortus* large ruminants and wildlife, *B. suis* infects pigs, and *B. canis* infects dogs (1, 2); however, potential infections in non-preferred hosts are possible and these species may act as reservoir hosts (3–5). Brucellosis causes abortions in animals and fever, fatigue, and loss of fertility in humans. It is found worldwide, especially in developing and tropical countries, whereas some countries (i.e., North and Central Europe, Australia, New Zealand, Japan, and Canada) are considered free in domestic livestock (6, 7). Human brucellosis is more prevalent in countries where animal brucellosis is endemic, or in non-endemic countries, when people return from endemic areas after exposure (8–10). *B. melitensis* infections affect approximately 400,000–12,500,000 humans, annually, particularly in South-Eastern Europe, the Middle East, and Central Asia (7, 11, 12). In animals, brucellosis is mainly transmitted by direct or indirect contact (*via* contaminated environment/fomites), whereas transmission to humans is mainly by ingestion of contaminated unpasteurized milk products or by direct exposure. After rabies, brucellosis is the second most transmissible zoonotic disease worldwide (13, 14). Serology remains the main diagnostic tool for brucellosis (e.g., Rose Bengal plate test (RBPT), serum agglutination test (SAT), complement fixation test (CFT), enzyme-linked immunosorbent assay (ELISA), and milk ring test (MRT)). Brucellosis can also be diagnosed by molecular detection (i.e., PCR). Isolation remains the golden standard for diagnosis but requires advanced laboratory and biosafety levels (e.g., level 3). In addition to being a notifiable disease, brucellosis is also considered a bio-threatening (category B) agent (15, 16).

## Animal Production in Pakistan

Pakistan is a South Asian country with an agriculture-based economy where the livestock subsector plays an integral role in agriculture. In 2019, the estimated livestock population of the country was 90.8 million cattle and buffaloes, 109.4 million small ruminants (sheep and goats), 1.1 million camels, and 6.1 million equines (17). This sub-sector contributed to 60.6% of the value of the agriculture sector and 11.7% of the national Gross Domestic Product (GDP) in 2019–20. Pakistan consists of seven administrative units, i.e., Punjab, Sindh, Balochistan, Khyber Pakhtunkhwa (KPK), Gilgit-Baltistan, Azad Jammu and Kashmir (AJK), and Islamabad Capital Territory (ICT). The majority of cattle and buffaloes is distributed around the irrigated areas of the country (i.e., Punjab and Sindh), whereas small ruminants are mainly distributed in the arid and semiarid areas (e.g., Baluchistan, KPK, Gilgit-Baltistan, and AJK). There is a strong animal-human relationship in Pakistan as owning livestock is not only a source of ready income but is also a symbol of financial status for institutions and individuals.

Brucellosis is considered endemic in Pakistan and has been reported in domestic ruminants (e.g., cattle, buffaloes, sheep and goats, and camels), non-ruminants (e.g., equines and dogs), wildlife, and humans. Brucellosis causes economic losses to the farmers in Pakistan, including the cost of treatment, fetal losses, infertility, reduced milk production, prolonged calving intervals, and losses due to the culling of the animals (18). To our knowledge, information concerning economic losses related to brucellosis in Pakistan is not available, but losses were estimated at US $3.4 billion for dairy cattle and US $58.8 million for active surveillance programs in India (19–21).

Pakistan produced 61.7 million tons of milk in 2019 and is ranked the fourth largest milk-producing country in the world. Approximately, 98% of the milk is marketed as raw milk, and the milk transport and storage practices are antiquated outside of large urban areas. Vaccination and treatment of brucellosis in animals are limited because of safety issues and are primarily practiced on animals of high economic value (21). Accidental contamination of milk (by vaccination or infection) not only poses negative economic effects on farmers but also is a public health threat. The purpose of this review was to review more recent data (previous 11 years) on brucellosis to emphasize the need for awareness programs, and to identify underlying problems related to disease control in Pakistan. Eliminating the shortcomings in current efforts will be beneficial in controlling brucellosis in Pakistan with a “One Health” perspective.

## Literature Search and Inclusion/Exclusion Criteria

A literature search was done online by using the keywords “brucellosis, *Brucella*, Pakistan” on Google Scholar (Google LLC, Mountain View, California) search bar in March 2021. The search was limited to the past 11 years (2011–2021) to generate contemporary data on brucellosis epidemiology in animals and humans in Pakistan. In total, 1,720 findings were scrutinized for being relevant, whereas duplicates, conference abstracts, reviews, and non-English articles were excluded. Authors of manuscripts with incomplete information or disclarity were contacted, and manuscripts were included only if queries were addressed. Overall, 72 peer-reviewed articles were selected for inclusion in the analysis. Information regarding geographical areas, host species, type of diagnostic tests used, and seroprevalence are presented in Tables 1, 2. For ease of understanding, the authors preferred to describe the seroprevalence by RBPT in the text.

**Table 1:** Animal brucellosis in Pakistan.

**Reference**	**Year of study**	**Location**	**Host**	**Serology**			
				**RBPT**	**SAT**	ELISA (i c., m)^*^	**MRT**
**Punjab**							
(22)	2018	Rajanpur	Buffalo	5.2% (13/259)	-	2% (5/250), i	-
			Cattle	4.4% (11/250)	-	1.2% (3/250), i	-
(23)	2013	Rawalpindi	Cattle and Buffaloes	4.6% (48/1052)			
		Attock		5.7% (61/1063)			
(24)	2011	Lahore	Cattle	52.3% (69/132)			
		Gujranwala		68.8% (33/48)			
		Okara		62.5% (15/24)			
		Hafizabad		0% (00/09)			
		Muzaffar Garh		66.7% (18/27)			
(25)	2013	Faisalabad	Horses	20.13% (62/308)	16.23% (50/308)		
(26)	2013	Layyah	Sheep	7% (27/384)	0% (0/384)	0% (0/384)	
(27)	2015	Organized and private livestock farms of Punjab	Goats	35.1% (99/282)	9.57% (27/282)	6.73% (19/282)	6.73% (19/282)
			Sheep	8.95% (117/1306)	2.14% (28/1306)	1.76% (23/1306)	1.91% (25/1306)
			Cattle	10.32% (48/475)	7.57% (36/475)	6.31% (30/475)	6.94% (33/475)
			Buffaloes	12.73% (27/212)	8.49% (18/212	8.01% (17/212)	8.01% (17/212)
(28)	2016	Hafizabad	Cattle	63.64% (42/66)		57.58% (38/66)	
(29)	2018	Okara	Buffaloes			18% (9/50)	14% (7/50)
		Jhang				12% (6/50)	8% (4/50)
(30)	2019	Gujranwala	Cattle	28.9% (111/384)		27.86% (107/384)	
(31)	2020	Organized Livestock Farns	Cattle	2.15% (9/419)		1.91% (8/419)	
			Buffaloes	5.62% (23/409)		4.64% (19/409)	
(32)	2019	Faisalabad	Stray and working dogs	9.2% (8/87)	10.3% (9/87)		
		Bahawalpur		63.8% (60/94)	0% (0/94)		
(33)	2020	Organized semen production units	Cattle	0% (0/118)		1.69% (2/118)	
			Buffaloes	0% (0/139)		2.14% (3/135)	
(34)	2020	Narowal	Cattle	24.6% (16/65)		24.6% (16/65)	
**Punjab**							
			Buffaloes	13.04% (9/69)		17.4% (12/69)	
		Gujranwala	Cattle	15.7% (11/70)		18.5% (13/70)	
			Buffaloes	12.5% (8/64)		12.5% (8/64)	
		Gujrat	Cattle	16.4% (12/73)		16.4% (12/73)	
			Buffaloes	16.3% (10/61)		16.3% (10/61)	
(35)	2020	Kasur	Cattle	3.03% (2/66)			
			Buffaloes	3.45% (3/55)			
		Faisalabad	Cattle	5% (2/40)			
				**RBPT**	**SAT**	ELISA (i c., m)^*^	**MRT**
			Buffaloes	5.61% (5/89)			
		Lahore	Cattle	4% (2/50)			
			Buffaloes	6.25% (2/32)			
		Okara	Cattle	4% (1/25)			
			Buffaloes	3.3% (2/60)			
(36)	2020	Sahiwal	Horses and Jackasses	27.1% (48/177)			
		Khanewal		25.8% (16/62)			
		Okara		15.3% (32/209)			
(37)	2020	Faisalabad	Mules	9.1% (2/22)	9.1% (2/22)		
			Donkeys	4.38% (7/160)	3.75% (6/160)		
(38)	2020	Organized livestock farms, Faisalabad and Okara	Goats	19.5% (73/374)	3.2% (37/374)		
			Sheep	7.1% (61/865)	9.9% (28/865)		
(39)	2021	Sargodha	Cattle	12% (12/100)		12% (12/100)	
		Sahiwal		14% (14/100)		14% (14/100)	
		Chiniot		12% (12/100)		12% (12/100)	
**Khyber Pakhtunkhwa (KPK)**							
(40)	2017	Kurram	Cattle	6.08% (9/148)		4.7% (7/148)	
			Buffaloes	8.57% (9/105)		4.8% (5/105)	
			Goats	5.6% (9/160)		3.1% (5/160)	
			Sheep	3.2% (5/154)		1.9% (3/154)	
(41)	2018	Bannu	Cattle	9.1% (7/77)	25.97% (20/77)		
			Buffaloes	13.04% (3/23)	30.4% (7/23)		
			Humans	5.69% (7/123)	26.83% (33/123)		
**Sindh**							
(42)	2014	Hyderabad	Cattle	25% (125%500)	23.2% (116/500)	11.8% (59/500)	
(43)	2015	Karachi	Cattle	17.5% (35/200)			
(44)	2017	Thatta	Camels	18.18% (6/33)	18.18% (6/33)	6.06% (2/33)	
		Badin		12.12% (4/33)	12.12% (4/33)	9.09% (3/33)	
		Tharparkar		32.35% (11/34)	32.35% (11/34)	23.5% (8/34)	
**Balochistan**							
(45)	2016	Turbat	Goats	2% (3/150)	1.33% (2/150)		
			Sheep	2.67% (4/150)	2% (3/150)		
(46)	2016	Loralai	Cattle	3.5% (14/400)	1.75% (7/400)		
(47)	2012	Quetta	Cattle				19.76% (17/86)
			Buffaloes				0% (0/114)
(48)	2011		Cattle	3.95% (16/405)		5.9% (24/405)	
			Buffaloes	2.1% (8/375)		0.26% (01/375)	
**Islamabad Capital Territory (ICT) and Azad Jammu Kashmir (AJK)**							
(49)	2017	ICT, Rawalpindi	Cattle	8.3% (5/60)		6.6% (4/60)	
			Buffaloes	1.6% (1/60)		1.6% (1/60)	
(50)	2013	Potohar Plateau	Cattle	5.01% (20/399)	4.76% (19/399)		
(51)	2013	Bhimber	Goats	13.33% (20/150)	11.33% (17/150)^*^		

* *(I, indirect; c, competitive; m, milk)*.

**Table 2:** Human brucellosis in Pakistan.

**Reference**	**Year**	**Province/territory**	**Area**	**Target group**	**RBPT**	**SAT**	**ELISA (i, c)s**
(52)	2013	**Punjab**	Potohar Plateau of Punjab	Occupationally exposed humans	6.87% (18/262)		
(53)	2016		Faisalabad	Occupationally exposed humans		38.94% (37/95)	
(54)	2016		Rawalpindi	Pregnant women	5.8% (25/429)		
(55)	2019		Rawalpindi	Febrile patients	10.7% (28/261)		
(56)	2014	**Khyber Pakhtunkhwa (KPK)**	Peshawar	Hospital outdoor		36.4% (455/1250)	60% (273/455) suffered from acute brucellosis and other 40% (182/455) from chronic brucellosis
(57)	2017		Karak	Humans		6% (12/200)	
(40)	2017		Kurram	Humans			3.04% (6/197)
(41)	2018		Bannu	Humans	6.84% (5/73)	24.6% (18/73)	
(58)	2017		Sawat	Humans		3.66% (11/300) 2% (6/300)	
(59)	2021		Malakand	Human females		18.42% (56/304)	27.47%
(55)	2019	**ICT**		Febrile patients	9.2% (17/185)		
(51)	2013	**Bhimber, AJK**		Humans	9.33% (14/150)	7.33% (11/150)	
						6.0% (9/150)	

* *(I,indirect; c,competitive)*.

## Diagnosis of Brucellosis

Serology has been a preferred choice for the diagnosis of brucellosis in Pakistan. The most common serologic test used in Pakistan by frequency is RBPT, SAT, MRT, and indirect-ELISA. The use of PCR or *Brucella* isolation techniques occurred less frequently. RBPT is commonly used for the screening of sera because it is inexpensive. ELISA is often used as a complementary test for RBPT-positive sera, although it has been used as a single screening test at some standardized laboratories (60). Standard SAT and its modifications have been used in KPK and Punjab. These three tests are highly sensitive with lower specificity. For lactating animals, indirect-milk-ELISA and MRT methods have been used (Table 1). Both conventional and real-time PCR-based *Brucella*-DNA (genus and species-specific) detection and differentiation methods have been used in Pakistan. The use of isolation and microbiological characterization of brucellae is increasingly applied but still not widely practiced because of the lack of appropriate laboratories, trained personnel, and risk of laboratory-acquired infections; however, both *B. abortus* and *B. melitensis* have been identified by molecular/microbiological techniques (61, 62). Data from advanced molecular typing techniques (e.g., SNP typing, MLVA, MLST, and antimicrobial susceptibility testing) are rare (63, 64).

## Brucellosis in Animals

**Figure 1:**
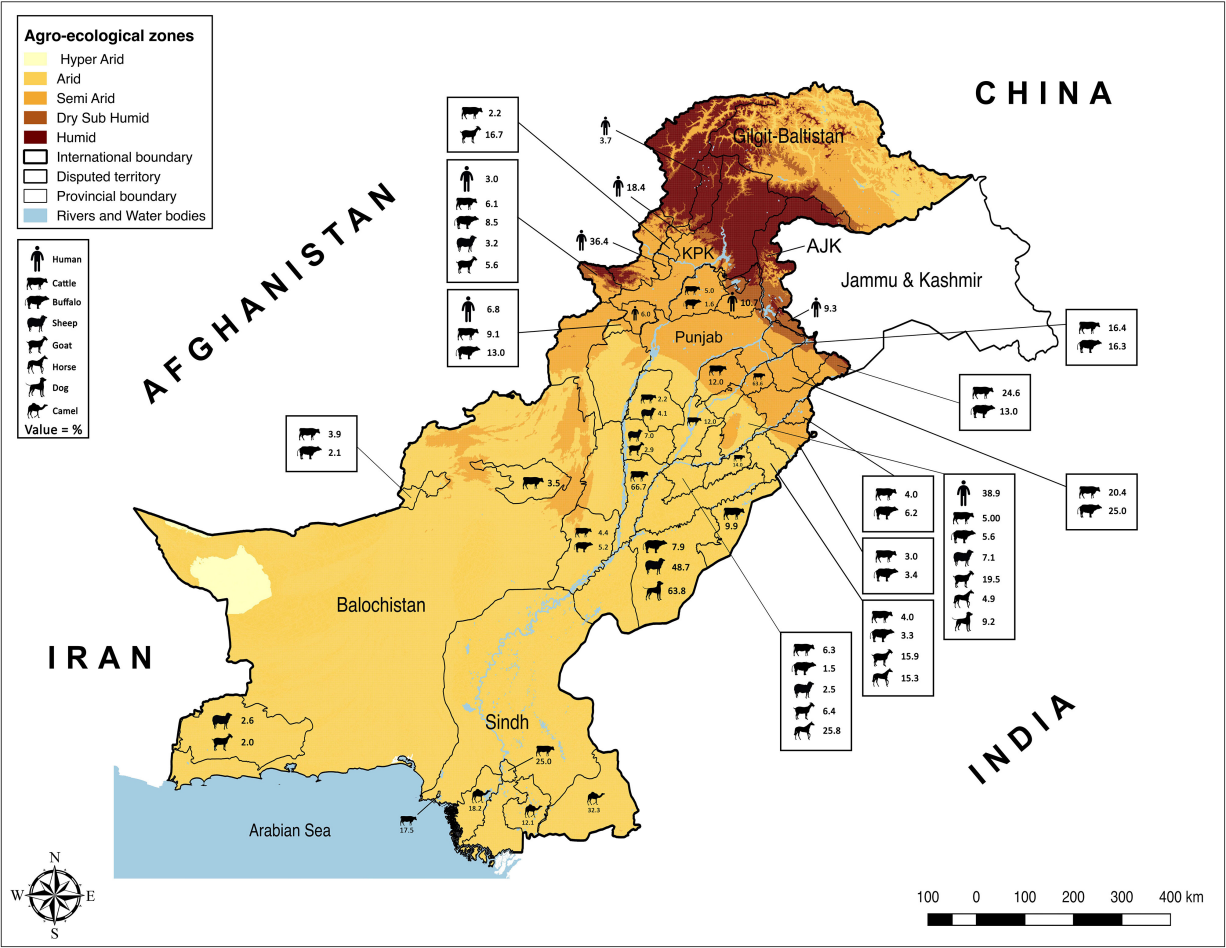
Animal and human brucellosis in Pakistan.

Punjab is the province with the largest population of humans and livestock. The fertile river basins and the monsoon rains (July-September) provide good conditions for agriculture production. Weather remains extremely varying from foggy winters (−2 ± 8°C) to hot summers (46 ± 8°C). Because of large numbers of both conventional and intensive farming systems and comparatively better veterinary diagnostic and surveillance systems, reports of brucellosis are highest in this province (Table 1). Estimates of brucellosis from this province are highly variable depending upon the type of the diagnostic test used, animal species, farming system, and environmental factors [e.g., 0–68.8% in cattle and buffaloes, 7.1–35.1% in sheep and goats, 4.38–20.13% in horses and donkeys, and 0–10.3% for bovine (*B. abortus*), and 9.2–63.8% for canine (*B. canis*) brucellosis in dogs]. Brucellosis has been reported in all types of animal farming systems (i.e., conventional, intensive, and smallholdings) (Figure 1) (22–25, 27, 28, 30, 32–39, 65–67). Higher disease prevalence was reported in confined farming systems as compared with free-ranging livestock populations. *Brucella*-DNA was also detected in soil along ancient trade routes in this province (68).

Sindh is the province with the second-highest human and livestock populations and the third-largest by land size. It comprises Thar Desert in the north to fertile Indus basin in the middle and is bordered by the Arabian Sea in the south. In this province, brucellosis is reported predominantly in bovines and camels of Karachi and Hyderabad, with no reports of sheep and goats (Figure 1). Seroprevalence ranged between 17.5 and 25% in cattle and between 12.12 and 32.35% in camels (42–44).

Khyber Pakhtunkhwa is located in northwest Pakistan and ranks third for livestock and human populations. The topography is predominantly mountainous including the Hindu Kush and the Suliman ranges. Although small ruminant population are much larger than bovine populations in this province, brucellosis has been reported in both bovines and small ruminants (0–13.4% in cattle and buffaloes and 3.2–16.67% in sheep and goats) (40, 41, 57).

Balochistan is located in southwest Pakistan, is the largest province by land area and the least populated province. It has a mountainous landscape with hot summers and cold winters. Livestock populations are predominantly small ruminants rather than large ruminants with brucellosis reported in bovines and small ruminants. Seroprevalence estimates ranged between 0.26–5.9% in cattle and buffaloes and 2–2.67% in sheep and goats from districts Quetta and Turbat, respectively (Table 1) (45, 46).

The Islamabad Capital Territory is located in north Pakistan. Brucellosis has been widely reported in this area in bovine and small ruminant herds with seroprevalence ranging from 1.6–8.3% in cattle and buffaloes and 2.2–13.1% in sheep and goats (23, 65, 69). Gilgit-Baltistan is an administrative territory in the north of Pakistan comprising the Himalayas, Hindu Kush, and Karakoram mountains. Reports of brucellosis in cattle (10.93%) and wild animals have occurred in this territory (70). AJK is a self-governing administrative territory of Pakistan that encompasses the lower range of the Himalayan mountains. Seroprevalence of brucellosis in goats was reported to be 13.33% (Figure 1) (51, 71).

A total of 17 field isolates of *B. abortus* biovar 1 from cattle and buffaloes in Punjab and 1 isolate of *B. melitensis* from goats in KPK have been reported (61, 63). So far, *B. melitensis* has been confirmed only by real-time PCR in cattle and buffaloes but not by isolation (62). In addition, *B. abortus* has been diagnosed only by real-time PCR in sheep and goats, horses, camels, and dogs without confirmation by isolates (32, 36, 69, 72). In addition, in wildlife, only anti-smooth-lipopolysaccharide (LPS)-*Brucella* (*B. abortus* and *B. melitensis*) antibodies have been reported without any PCR-based detection (73). Only one report was found describing the detection of canine brucellosis from Punjab by serology (32).

Epidemiologic variables influencing brucellosis prevalence have been studied to determine the risk. Higher disease prevalence was correlated with animal genetics as buffaloes, crossbred and exotic cattle have higher rates of brucellosis (23, 28, 40, 57, 63). It has been hypothesized that a specific allele on *nramp1* in Sahiwal cows increases brucellosis resistance, but data are limited so far (74). Female animals tested positive for brucellosis at a higher rate than males (50, 65). This observation may be influenced by the fact that (i) fewer bulls than cows are kept on farms and (ii) increasing use of artificial insemination at the farms. Greater risk for brucellosis was also associated with maturity (>4 years of age), the number of pregnancies completed, increased frequency of contact with other animals (65, 75), retention of fetal membranes, and a history of abortion (30, 65, 75, 76). Other risk factors included the following: geographical location, management system (institution-owned, private, or general population), and herd size (62, 65, 76). The most common cause of brucellosis infection at private-owned farms was a breach in biosecurity (i.e., the introduction of carrier animals without screening) (31). The infection often remains unidentified until abortions occur or until animals are tested positive for brucellosis in screenings.

## Brucellosis in Humans

To our best knowledge, in Pakistan, the first human brucellosis study was reported in 1979 (77). Currently, it has been reported that among all provinces, the highest numbers of patients were from Punjab (5.8–10.7% at hospital outdoor and 6.87–38.94% in occupationally exposed) and KPK (2–36.4% at hospital outdoor) but is still considered highly underreported and misdiagnosed (52, 53, 56, 58, 62, 78). Brucellosis generally presents as undulant fever or as chronic malaise (Table 2) (55, 79). Human patients without abortion or orchitis are treated for general febrile malaise and brucellosis is suspected when patients do not respond to routine treatments (80). The patients are often serologically positive but are negative by PCR or culture if these two approaches are performed. Delays in the onset of appropriate treatment often cause more severe or chronic disease symptoms with cardiac, intestinal, nervous, and/or pulmonary complications. Antimicrobials most frequently used to treat human brucellosis are tetracyclines, aminoglycosides, and fluoroquinolones (80). Both *B. abortus* and *B. melitensis* have been confirmed by PCR in human patients (55, 57). So far, no isolate has been reported from humans. It is estimated that occupational exposure, especially among veterinarians and farmers, and raw milk intake are the greatest risk factors for human brucellosis (45, 54, 78, 81). As humans are dead-end hosts, the presence of brucellosis in humans indicates a disease burden in animals. Thus, controlling the disease in animals is the most economic approach to address brucellosis in humans (9). Despite governmental initiatives, knowledge of farmers on the risks of brucellosis to date remains low in rural areas (81).

## Discussion

Brucellosis remains an important disease in domestic livestock and humans in Pakistan (Tables 1, 2). The review of the studies indicated a higher brucellosis prevalence in the areas with high human population, intensive animal farming, high animal-human interaction (e.g., livestock markets, slaughterhouses, livestock breeding and dairy farms, semen production units, and veterinary hospitals), and livestock trade routes (Figure 1). In addition, consuming unpasteurized dairy milk and neglecting protective measures during handling animals and animal products at parturition/abortion, milking, and/or medication time appeared to be contributing factors. As the disease can stay asymptomatic in animals and remain undiagnosed, such practices pose a high public-health safety risk to the workers and associated people. The main cause for infections in livestock is the breach of biosecurity by the introduction of infected animals to disease-free herds (31). Animals are not screened for infectious diseases and/or are not quarantined before entry at most dairy farms. In addition, biosecurity measures of the farm premises often do not extend to other domestic animals (e.g., camels) or wildlife (32, 73). Their potential role is often ignored by the workers. In remote areas, the birth/aborted products of the animals are seldomly buried or disposed-off properly and are often simply drained into the sewage or nearby canals or thrown away openly. These disposals are then accessible to stray or wild carnivores. Even the farm dogs are usually fed with condemned milk and dairy products produced at the farm. Brucellae are killed by heat (60°C for 10 min), ultraviolet radiation, and low-level disinfectants, but can survive up to several weeks in the presence of organic matter and soil (82). The traces of *Brucella*-DNA found in the soils of Punjab, where animal-human interaction was at maximum, confirm its serious menace toward public health safety and environment (68). Hence, there is the need for a “One Health” approach in controlling this disease.

The authors summarize the major obstacles in brucellosis prevention and control programs in Pakistan as follows: (i) poor implementation of strict biosecurity and standard operation procedures (SOPs) at livestock farms, (ii) poor disease surveillance and limited access to diagnostic laboratories in some areas, (iii) lack of personal protection when handling diseased animals, carcasses, or diagnostic specimens, (iv) lack of communication between human patients and medical personnel regarding disease symptoms, (v) lack of maintenance of data records on animal sales, movements, disease status, and vaccination at farms, (vi) incorrect knowledge on the effects of immunization, treatment and culling procedures/policies by farmers, and (vii) lack of appropriate laboratories for the isolation, identification, and typing of isolates.

Brucellosis is one of the priority zoonotic diseases of the national infection control and eradication program of the National Institute of Health (NIH), Islamabad, Pakistan. A national brucellosis control program was implemented in 2018–2023 that included capability enhancement of national and provincial laboratories, veterinary and human hospitals (26). The program implemented the use of animal tagging, register-based records, serologic monitoring, and farm biosecurity measures to control brucellosis (66). Continued strengthening of regulatory infrastructure (e.g., a laboratory network), additional training of human resources, and greater interdepartmental coordination were proposed for the brucellosis control program. Outbreaks of brucellosis can be prevented by purchasing animals from brucellosis-free herds, quarantine and testing animals before introduction into the herd, and using semen from brucellosis-free bulls. Quarantine measures for infected animals and herds must be implemented. Vaccines should be used according to their safety protocols and local veterinary legislation. An important drawback is that most serological tests are unable to differentiate between vaccinated and infected animals. Human brucellosis can be overcome by controlling animal brucellosis and ensuring the availability of pasteurized dairy milk for human consumption (21). There are still multiple challenges at the human-animal-environment interface for controlling brucellosis in the country. Two of the greater challenges include the lack of continuous disease surveillance programs and lack of coordination between agricultural/environmental, livestock and health departments.

## Conclusion

Brucellosis remains a persisting and challenging health hazard in Pakistan. Exotic dairy cattle producing high quantities of milk at intensive farms need higher standards of biosecurity. Occupationally exposed individuals are more prone to contract brucellosis and should adopt protective measures including regular screening. Current serological tools and surveillance systems must be updated and standardized especially considering rough strains of *Brucella*. Implementation of PCR diagnostics should be encouraged. Isolation techniques should be implemented and correspondingly the number of regional laboratories capable of safely handling samples and resulting isolates need to be increased. The potential of wildlife and other species to serve as reservoirs for brucellosis should be assessed. A nationwide vaccination program considering regional conditions/legislation should be implemented. Databases on national and regional brucellosis testing are needed. Education programs for farmers and veterinarians should be initiated with reference to the “One Health” concept to secure the acceptance of measures implemented to control brucellosis. Food safety regulations and consumer education should be strengthened. Finally, the human health sector needs to improve diagnostic facilities of brucellosis, increase patient education, and must effectively treat brucellosis cases.

## Author Contributions

TJ, AUK, and SS conceived the idea for this study. TJ, AUK, MI, and QU did literature search and collection. TJ wrote the manuscript and MZS, MAK, and WA helped in technical revisions. MHH and AR produced the map. MS, FM, SA, AS, IK, MKM, HN, and SS critically reviewed the manuscript. All authors agreed to the final version.

## Conflict of Interest

The authors declare that the research was conducted in the absence of any commercial or financial relationships that could be construed as a potential conflict of interest.

## Publisher's Note

All claims expressed in this article are solely those of the authors and do not necessarily represent those of their affiliated organizations, or those of the publisher, the editors and the reviewers. Any product that may be evaluated in this article, or claim that may be made by its manufacturer, is not guaranteed or endorsed by the publisher.
